# Changes in emergency psychiatric consultations in time of COVID-19: a retrospective observational study in the Verona Academic Hospital over the two pandemic years 2020–2021

**DOI:** 10.1186/s12873-023-00788-9

**Published:** 2023-02-15

**Authors:** Luca Bodini, Chiara Bonetto, Antonio Maccagnani, Antonio Bonora, Enrico Polati, Giorgio Ricci, Ciro Paolillo, Francesco Amaddeo, Antonio Lasalvia

**Affiliations:** 1 Section of Psychiatry, Department of Neuroscience, Biomedicine and Movement Sciences, University of Verona, Verona, Italy; 2 UOC Pronto Soccorso, Policlinico “G.B. Rossi”, DAI Emergenza e Terapie Intensive, Azienda Ospedaliera Universitaria Integrata (AOUI) di Verona, Verona, Italy; 3 Department of Anaesthesia and Intensive Care B, University of Verona, DAI Emergenza e Terapie Intensive, Azienda Ospedaliera Universitaria Integrata (AOUI) di Verona, Verona, Italy; 4 UOC Pronto Soccorso, Ospedale Civile Maggiore, DAI Emergenza e Terapie Intensive, Azienda Ospedaliera Universitaria Integrata (AOUI) di Verona, Verona, Italy; 5 UOC Psicosomatica e Psicologia Medica, Azienda Ospedaliera Universitaria Integrata (AOUI) di Verona, Verona, Italy; 6 UOC Psichiatria, Azienda Ospedaliera Universitaria Integrata (AOUI) di Verona, Verona, Italy

**Keywords:** Coronavirus, COVID-19, Mental health, Lockdown, Outbreak, Emergency department

## Abstract

**Background:**

During the first months of the COVID-19 pandemic, local health authorities in most Italian regions prescribed a reduction of ordinary outpatient and community mental health care. The aim of this study was to assess the impact of the COVID-19 pandemic on access to the emergency departments (ED) for psychiatric consultation in the pandemic years 2020 and 2021 compared to 2019.

**Methods:**

This is a retrospective study conducted by using routinely collected administrative data of the two EDs of the Verona Academic Hospital Trust (Verona, Italy). All ED psychiatry consultations registered from 01.01.2020 to 31.12.2021 were compared with those registered in the pre-pandemic year (01.01.2019 to 31.12.2019). The association between each recorded characteristic and the year considered was estimated by chi-square or Fisher’s exact test.

**Results:**

A significant reduction was observed between 2020 and 2019 (-23.3%) and between 2021 and 2019 (-16.3%). This reduction was most evident in the lockdown period of 2020 (-40.3%) and in the phase corresponding to the second and third pandemic waves (-36.1%). In 2021, young adults and people with diagnosis of psychosis showed an increase in requests for psychiatric consultation.

**Conclusions:**

Fear of contagion may have been an important factor in the overall reduction in psychiatric consultations. However, psychiatric consultations for people with psychosis and for young adults increased. This finding underlines the need for mental health services to implement alternative outreach strategies aimed to support, in times of crisis, these vulnerable segments of the population.

## Background

Italy was the first nation among Western countries to be affected by the COVID-19 outbreak. Due to the rapid spread of the pandemic within the country, on March 8th, 2020, the Italian government established stringent containment measures in Lombardy, Veneto, and some neighbouring provinces of Emilia-Romagna. On March 11th, 2020, the Italian government imposed a nationwide lockdown. This extraordinary containment measures restricted movement of all people across the national territory, except for work or health reasons or in an exceptional case of necessity. Starting from mid-March 2020, activities within hospitals in the most affected regions, such as Lombardy, Veneto and Emilia Romagna, underwent a rapid and profound reorganization in order to preserve beds and staff for COVID-19 patients [1, 2].

As a result, many hospital wards had to reorganize their activity, thus becoming COVID-19 wards. In this context, mental health care also underwent significant changes: some psychiatric inpatient units across the country were closed and a significant proportion of psychiatrists, mental health nurses, and other health-care workers were transferred to new COVID-19 wards [3].

During the lockdown period, local health authorities in most Italian regions, following national regulations, prescribed a reduction of ordinary outpatient and community mental health care; only mental health care for most urgent cases was ensured. Starting from mid-March 2020, day-care facilities for psychiatric patients were temporarily closed (in most regions they were reopened only in June/July 2020), whereas patients receiving residential care were confined within facilities with no possibility to follow outdoor rehabilitative interventions. In addition, the lockdown and the subsequent quarantine measures significantly impacted on the psychological status of the general population, thus leading to the onset of new mental disorders or to the exacerbation of pre-existing sub-threshold psychiatric conditions [4–6].

Taken together, the reduction of outpatient mental health care established by national and regional containment measures and the increase in the incidence of mental disorders in the general population might have led to increased numbers of access to emergency departments for psychiatric consultations during the first pandemic year.

On the contrary, research carried out in the first lockdown period both in Italy and in other countries reported a decrease in emergency department (ED) psychiatric consultations, with a reduction varying between 31 and 52% according to different studies [7–12]. The overall reduction of ED psychiatric consultations was mainly due to the reduction of consultations for anxiety and depressive disorders, whereas consultations for psychotic disorders did seem to have shown a substantial reduction [13].

Within this scenario, it is necessary to understand the dynamics that might have affected ED psychiatric consultations during the different pandemic phases. Studies conducted so far only considered limited periods of time, as they generally focused on the lockdown period or the post-lockdown periods of 2020 [7]. No study has so far monitored ED psychiatric consultations throughout the first (2020) and the second pandemic year (2021). To fill this knowledge gap, the aim of the following study is to evaluate the changes in ED psychiatric consultations for the years 2019, 2020 and 2021, and to assess whether significant differences exist stratifying by gender, age, and psychiatric diagnoses. In addition, by using the Interrupted Time Series Analysis (ITSA) technique, we aimed to assess whether restrictive measures had some role in changing the trend of ED psychiatric consultations over the years considered.

## Methods

### Setting

This study was conducted within the Verona Academic Hospital Trust [Azienda Ospedaliera Universitaria Integrata (AUOI) of Verona], the second largest hospital in Italy in terms of bed numbers (1384 ordinary and 138 day-hospital beds), and the fifth largest in terms of admissions. AOUI is a tertiary hospital trust composed of two hospitals, “Ospedale Civile Maggiore” and “Policlinico G.B. Rossi”, located respectively in the northern and southern part of the city of Verona (Veneto region, north-east Italy). Each hospital has its own specific ED. In the pre-pandemic era, the overall number of yearly accesses to both EDs was around 100,000.

### Study design and data collection

We conducted a retrospective observational study using an electronic administrative database (the ED information system) that collects routine information of all the emergency visits occurring within the two EDs operating in the Verona Academic Hospital Trust. For the specific study aims, we selected all ED psychiatric consultations for the adult population (≥ 18 years) that were registered by the ED information system in 2019, 2020 and 2021. For each ED psychiatric consultation, the following variables were collected: admission date; sex; age group; admission to any hospital ward for psychiatric reasons; primary psychiatric diagnosis. Psychiatric diagnoses were made on clinical basis by ED physicians (together with psychiatrists called for the consultation) using the International Classification of Diseases version 9—Clinical Modification (ICD-9-CM) codes. For the purposes of analysis, ICD-9-CM codes were grouped within macro categories (anxiety disorders; schizophrenia and other psychotic disorders; depression and mood disorders; adjustment disorders; personality disorders; suicide and intentional self-inflicted injury; poisoning by medications and drugs; delirium, dementia, amnestic and other cognitive disorders) using the Agency for Health Care Quality and Research (AHRQ) Clinical Classifications Software (CCS) for ICD-9-CM [11].

Data were anonymized and all patient identification details were removed by an independent administrator before data extraction to secure patients’ personal information in accordance with Data Protection Act (EU Regulation 679/2016). Therefore, research team members had no access to personal patient data. Informed consent to participate was waived by the Ethics Committee of the Provinces of Verona and Rovigo due to the retrospective nature of the study and to the fact the patient data were anonymized/de-identified. The study was approved by the Ethics Committee of the Provinces of Verona and Rovigo (approval No. 14892; March 15, 2021).

### Time period

This study analysed three consecutive years: 2019 (the “pre-pandemic year”, considered as the “control” condition), 2020 (the “first pandemic year”), 2021 (the “second pandemic year”). Within the two pandemic years, we identified some periods defined based on the pandemic stage and characterized by specific containment measures as enforced by the Italian legislation. Specifically, for 2020 we defined the following periods: (1) the “pre-lockdown phase” (January 1–March 8); (2) the “lockdown phase” (March 9–May 3); (3) the period of “loosened restrictions” (May 4–June 14); (4) the period of “relaxing restrictions” (June 15–October 12); (5) the period of “new restrictive measures due to the second pandemic wave” (October 13–December 31). For 2021, we identified the following periods: (1) the period of “restrictions due to the second and third pandemic wave” (January 1–April 25); (2) the period of “loosened restrictions” (April 26–June 6); (3) the period of “relaxing restrictions” (June 7–December 17); (4) the period of “new restrictions due to the fourth pandemic wave” (December 18–December 31).

### Data analysis

Descriptive statistics were performed, with ED psychiatric consultations presented as absolute numbers and percentages. Percentage scores from the pre-pandemic year 2019 were calculated for 2020 and 2021. Confidence intervals at 95% were estimated by assuming a Poisson distribution for the number of consultations. The association between each characteristic (gender, age bands, admission to a psychiatric ward, diagnosis) and the year was estimated by chi-square or Fisher’s exact test. Using the ‘itsa’ Stata command, a monthly interrupted time-series analysis (ITSA) with a single-group design and multiple treatment periods was estimated to assess whether the introduction of restrictive measures (9 March 2020: lockdown for the first pandemic wave; 13 October 2020: new restrictive measures for the second and third pandemic waves) resulted in a shift in the level and trend over time of ED psychiatric consultations [14]. Newey-West standard errors were estimated to adjust the standard errors to handle possible heteroskedasticity and a maximum number of lags of 1 to handle any auto correlation.

All tests were bilateral at a significance level of 0.05. Analyses were performed by Stata 17 for Windows.

## Results

With respect to the pre-pandemic year (2019), the overall number of ED visits within the Verona Hospital Trust decreased by 23% in 2020 (from 99,829 in 2019 to 77,191 in 2020) and by 15% in 2021 (from 84,530 to 77,191).

As regards ED psychiatric consultations, changes occurring over the two pandemic years followed the same pattern observed for overall ED visits (see Table 1).

**Table 1:** ED psychiatric consultations: comparisons between 2020 and 2021 with 2019 by different pandemic periods

**Comparison between 2020 and 2019**					
**Period**	**Description**	**2019**	**2020**	**Change in 2020**–**2019**^**#**^	**Poisson 95% CI**
		**n (%)**	**n (%)**	**%**	
1 Jan–8 Mar	Pre-lockdown	246 (20.3)	194 (20.9)	-21.1	(-27.7; -15.8)
9 Mar–3 May	Lockdown	181 (14.9)	108 (11.6)^↓^	-40.3	(-50.7; -31.6)
4 May–14 Jun	Loosened restrictions	163 (13.4)	120 (12.9)	-26.4	(-35.5; -19.1)
15 Jun–12 Oct	Relaxed restrictions	367 (30.4)	339 (36.4) ^↑^	-7.6	(-11.0; -5.1)
13 Oct–31 Dec	New restrictive measures for the second pandemic wave	255 (21.0)	169 (18.2)	-33.7	(-41.7; -27.0)
Whole period		1212	930	-23.3	(-26.1; -20.6)
**Comparison between 2021 and 2019**					
**Period**	**Description**	**2019**	**2021**	**Change in 2021–2019**^**°**^	**Poisson 95% CI**
		**n (%)**	**n (%)**	**%**	
1 Jan–25 Apr	Restrictions due to the second and third pandemic wave	391 (32.3)	295 (29.1)	-24.6	(-30.0; -19.9)
26 Apr–6 Jun	Loosened restrictions	155 (12.8)	99 (9.8) ^↓^	-36.1	(-46.9; -27.3)
7 Jun–17 Dec	Relaxed restrictions	618 (51.0)	585 (57.6) ^↑^	-5.3	(-7.5; -3.7)
18 Dec–31 Dec	New restrictions due to the fourth pandemic wave	48 (4.0)	36 (3.5)	-25.0	(-43.7; -12.9)
Whole period		1212	1015	-16.3	(-18.7; -14.1)

^#^(Number of consultations 2020–Number of consultations 2019)/Number of consultations 2019

^°^ (Number of consultations 2021–Number of consultations 2019)/Number of consultations 2019

Chi-square test for the distribution of the number of consultations by period and year: 2019–2020 *p* = 0.012; 2019–2021 *p* = 0.011

^↓^ adjusted residual < -1.96 (the number of consultations is significantly smaller than would be expected if the year and the period are independent)

^↑^ adjusted residual >  + 1.96 (the number of consultations is significantly larger than would be expected if the year and the period are independent)

With respect to the pre-pandemic year, ED psychiatric consultations also decreased by 23% in 2020. The greatest reduction was observed, as expected, during the lockdown period (40.3%), followed by the period of new restrictions due to the second pandemic wave (33.7%), whereas the reduction during the period of relaxing restrictions (broadly corresponding to the summer season) was negligible (7.6%) [95% CI (-11.0; -5.1)] (Table 1, upper part).

With respect to the pre-pandemic year, the ED psychiatric consultations in 2021 were also reduced, but to a lesser degree than 2020 (16.3%) [95% CI: (-26.1; -20.6) vs (-18.7; -14.1)]. The greatest reduction in ED psychiatric consultations in 2021 was observed in the period of loosened restrictions following the second and third pandemic wave (36.1%); a relevant reduction was also detected in the periods of new restrictions due to the fourth (25%) and the second pandemic waves (24.5%). No reduction in ED psychiatric consultations was detected during the second part of 2021, except for the last two weeks of December (Table 1, bottom part).

**Fig. 1:**
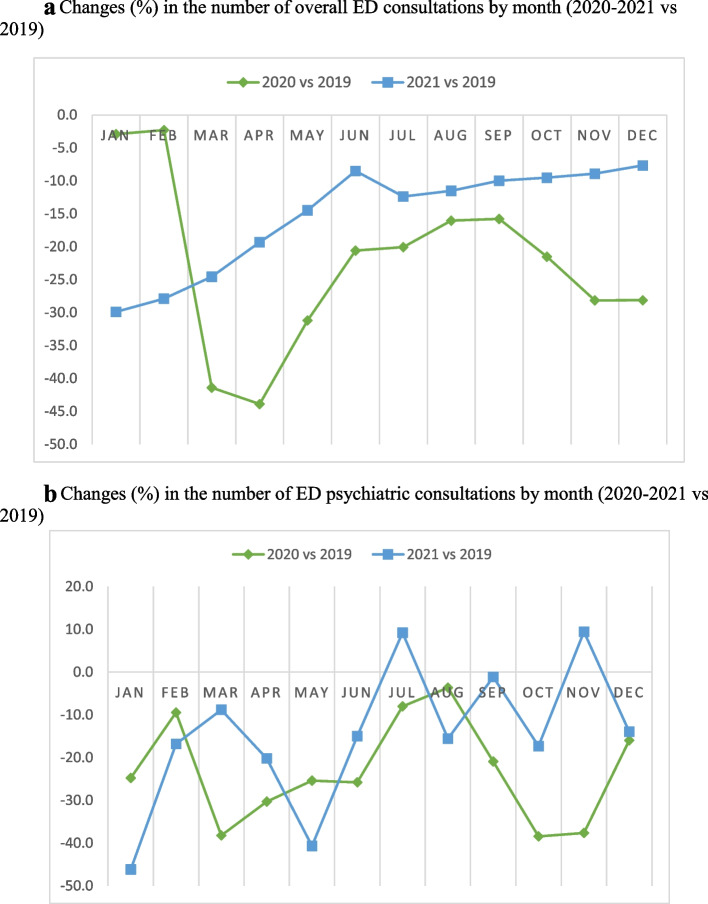
**a** Changes (%) in the number of overall ED consultations by month (2020–2021 vs 2019). **b** Changes (%) in the number of ED psychiatric consultations by month (2020–2021 vs 2019)

Figure 1a reports percentage changes in the number of overall ED visits by month. As compared with the pre-pandemic year, the number of ED visits over the 12-month period was reduced during 2020, with the greatest reduction in April (44%), corresponding to the mid-lockdown phase. During the summer season 2020, reduction in ED visits never exceeded 16%. The second pandemic year displayed a less pronounced trend of reduction of ED visits, progressively approaching a reduction of 8.5% on June. In December 2021 the reduction of ED visits was only of 7.7% with respect to the pre-pandemic year.

Figure 1b reports percentage changes between 2020 and 2019 and between 2021 and 2019 in monthly ED psychiatric consultations. As compared to the pre-pandemic year, the greatest reduction in ED psychiatric consultation was first detected in March 2020 (38%), followed by October–November 2020 (respectively, 38.5% and 37.6%); the reduction in ED psychiatric consultations remained evident throughout the first pandemic year, with the only exception being August when the reduction reached a negligible level (3.7%). As compared to the pre-pandemic year, in January 2021 the greatest reduction was observed in ED psychiatric consultations since the beginning of the pandemic (46%), whereas the number of ED psychiatric consultations did not significantly change during the second half of 2021.

Table 2 shows the distribution of psychiatric consultations by year and key patients’ characteristics (gender, age bands, admission to hospital, psychiatric diagnosis). No significant difference in the cohort composition was found between 2020 and 2019, while age and diagnosis significantly differed between 2021 and 2019, with a higher percentage of ED consultations for young patients (27.5% vs 22.3%) and patients with psychosis (22.9% vs 18.4%) in the second pandemic year.

**Table 2:** ED psychiatric consultations by socio-demographics and diagnosis by year (2019–2020-2021)

Characteristics	2019	2020	2021	*p*-value^*^ 2020 vs 2019	*p*-value^*^ 2021 vs 2019	Change in 2020 with respect to 2019^#^	Change in 2021 with respect to 2019^°^
	**N (%)**	**N (%)**	**N (%)**			**% (Poisson 95% CI)**	**% (Poisson 95% CI)**
**Gender**	(7 missing)	(4 missing)	(1 missing)				
Female	646 (53.6)	491 (53.0)	526 (51.9)	0.793	0.418	-24.0 (-28.1; -20.4)	-18.6 (-22.2; -15.4)
Male	559 (46.4)	435 (47.0)	488 (48.1)			-22.2 (-26.4; -18.5)	-12.7 (-16.0; -9–9)
**Age**							
18–30	270 (22.3)	222 (23.9)	279 (27.5) ^↑^	0.201	0.036	-17.8 (-23.6; -13.1)	+ 3.3 (+ 1.5; + 6.3)
31–50	435 (35.9)	304 (32.7)	328 (32.3)			-30.1 (-35.7; -25.2)	-24.6 (-29.7; -20.2)
51–70	370 (30.5)	312 (33.5)	295 (29.1)			-15.7 (-20.3; -11.9)	-20.3 (-25.4; -15.9)
> 70	137 (11.3)	92 (9.9)	113 (11.1)			-32.8 (-44.0; -24.0)	-17.5 (-26.1; -11.2)
**Admission**							
No	675 (55.7)	544 (58.5)	533 (52.5)	0.202	0.135	-19.4 (-23.0; -16.2)	-21.0 (-24.8; -17.7)
Yes	537 (44.3)	386 (41.5)	482 (47.5)			-28.1 (-33.0; -23.8)	-10.2 (-13.3; -7.7)
**Diagnosis**							
Anxiety	423 (34.9)	296 (31.8)	319 (31.4)	0.213	0.035	-30.0 (-35.7; -25.0)	-24.6 (-29.8; -20.1)
Psychosis	223 (18.4)	168 (18.1)	232 (22.9) ^↑^			-24.7 (-32.1; -18.6)	+ 4.0 (+ 1.8; + 7.7)
Mood	205 (16.9)	148 (15.9)	142 (14.0)			-27.8 (-36.0; -21.1)	-30.7 (-39.3; -23.6)
Suicide, poisoning	72 (5.9)	77 (8.3)	59 (5.8)			+ 6.9 (+ 2.3; + 16.2)	-18.1 (-30.9; -9.6)
Substance abuse	61 (5.0)	55 (5.9)	63 (6.2)			-9.8 (-21.4; -3.6)	+ 3.3 (+ 0.4; + 11.8)
Other	228 (18.8)	186 (20.0)	200 (19.7)			-18.4 (-24.9; -13.3)	-12.3 (-17.7; -8.2)

^#^ (Number of consultations 2020–Number of consultations 2019)/Number of consultations 2019

^°^ (Number of consultations 2021–Number of consultations 2019)/Number of consultations 2019

^*^ Chi-square or Fisher’s exact test

^↑^ adjusted residual >  + 1.96 (the number of consultations is significantly larger than would be expected if the year and the characteristic are independent)

^↓^ adjusted residual < -1.96 (the number of consultations is significantly smaller than would be expected if the year and the characteristic are independent)

Considering the percentage changes stratified by key characteristics, a reduction for all categories in all variables was observed in 2020 with respect to 2019. Specifically, females and males showed a similar reduction. As regards the age bands, both older (> 70 yrs.) and middle-aged patients (31–50 yrs.) displayed a 30% reduction (95% CIs overlapping). A 28% reduction was observed in admissions to hospital following ED psychiatric consultations. Regarding diagnostic composition, ED consultations for anxiety, psychosis and mood disorders showed a reduction during 2020, ranging from 25 and 30%; on the other hand, ED consultations for suicide/self-poisoning increased up to nearly 7% (95% CI 2.3–16.2). The year 2021 was also characterized by a reduction in almost all categories for the considered characteristics, but to a lesser extent than 2020. Interestingly, young adults showed an increase of up to 3% with respect to 2019. Moreover, psychosis had a 4% increase in 2021, whereas ED consultations for suicide/self-poisoning displayed an 18% reduction in 2021. Finally, ED consultations for substance abuse disorders increased by 3% in 2021.

As shown in Table 3, the starting level of the ED psychiatric consultations was estimated at about 109, and consultations appeared to decrease significantly prior to lockdown (9 March 2020) by 1.5 every month (*p* = 0.007).

**Table 3:** Interrupted time-series analysis to assess the effect of restrictive measures^*^ for monthly ED psychiatric consultations^#^

ED psychiatric consultations	Coefficient	Newey-West standard error	t	*p*-value	95% CI
Slope or trajectory of monthly consultations until the introduction of COVID-19 restrictions	-1.49	0.51	-2.90	0.007	(-2.53; -0.44)
Change in the level of consultations in the period immediately following the lockdown	-13.77	9.73	-1.42	0.167	(-33.64; 6.09)
Change in the monthly trend of consultations relative to the pre-lockdown period	2.43	2.69	0.90	0.375	(-3.07; 7.92)
Change in the level of consultations in the period immediately following the new restrictions	-11.84	13.62	-0.87	0.392	(-39.66; 15.99)
Change in the monthly trend of consultations relative to the lockdown period	0.98	2.83	0.35	0.730	(-4.80; 6.76)
Starting level of consultations	108.66	5.46	19.90	< 0.001	(97.51; 119.81)

^*^ First intervention: 9 March 2020 (lockdown for the first pandemic wave); second intervention: 13 October 2020 (new restrictive measures for the second and third pandemic waves)

^#^ No. of months: 36 (from January 2019 to December 2021)

In the first month after lockdown, a decrease in ED psychiatric consultations of 14 consultations emerged, although not statistically significant (*p* = 0.167). It was followed by an increase in the trend of consultations (relative to the pre-lockdown trend) of 2.4 consultations per month, but still not significant (*p* = 0.375). The posttrend estimation showed that, after the introduction of lockdown, ED psychiatric consultations did not change monthly (coefficient = 0.94, p = 0.722, CI = [-4.42, 6.30]).

The coefficients for the new restrictive measures period were compared with those of the lockdown period. In the first month of the new restrictions, there appeared to be a not significant decrease in ED psychiatric consultations of about 12 (*p* = 0.392). It was followed by an increase in the monthly trend of consultations (relative to the lockdown trend) of 1.0 consultations per month (*p* = 0.730), but it was not significant. The posttrend estimation showed that, after the introduction of the new restrictive measures, ED psychiatric consultations increased monthly at a rate of 1.92 (*p* = 0.002, CI = [0.75, 3.10]).

**Fig. 2:**
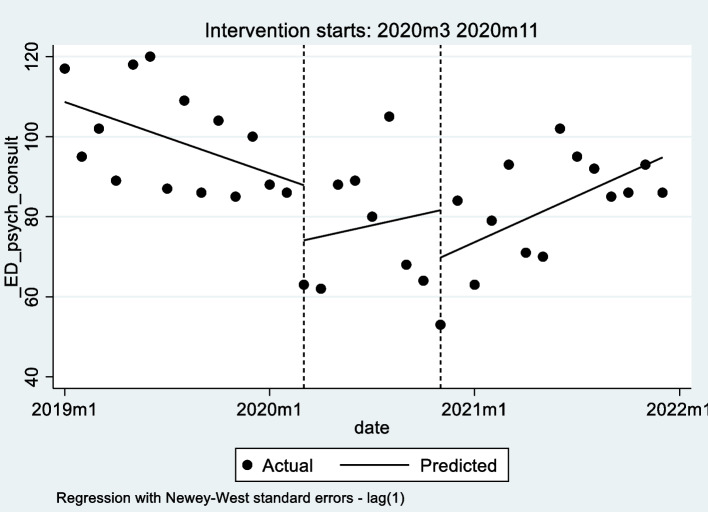
Monthly actual and predicted (by ITSA) ED psychiatric consultations in relation to COVID-19 restrictive measures^*^.^*^ First intervention: 9 March 2020 (lockdown for the first pandemic wave); second intervention: 13 October 2020 (new restrictive measures for the second and third pandemic waves)

Monthly actual and predicted ED psychiatric consultations in relation to COVID-19 restrictive measures are shown in Fig. 2.

## Discussion

Overall, we found a significant reduction (nearly one-quarter) of ED psychiatric consultations in the first pandemic year (2020) as compared to 2019. This finding parallels the reductions of overall ED visits both in our hospital and in other geographical areas [15–17]. Interestingly, the reduction of ED psychiatric consultations also occurred in the second pandemic year, even if to a lesser degree. We also found that the reduction of ED psychiatric consultations in the first pandemic year was mainly due to the reduction that occurred during the lockdown period. This finding is consistent with that reported by studies conducted in Italy [7, 10, 13] and in other nations [11, 12]. The reduction of ED psychiatric consultations during the lockdown may be explained in the light of the government restrictive measures that limited (or avoided) free movement across the national territory, together with the self-limitation of patients in referring to emergency departments due to the fear of contagion (indeed, over the first months of the pandemic, hospitals represented the main source of contagion).

The overall number of ED psychiatric consultations over the first pandemic year never returned to that of pre-pandemic level either when, following the significant reduction of infections and deaths from COVID-19, the most stringent restrictive measures were lifted. In this period, September 2020, our data shows an opposite trend with an increase in the number of admissions for psychiatric consultations. Most interestingly, despite this trend inversion, the overall number of psychiatric consultations also remained substantially lower than that of the pre-pandemic year during the second pandemic year (2021). Our study also found that, with respect to the pre-pandemic year, ED psychiatric consultations in 2021 were more frequent than expected for people aged 18–30 years. This seems a particularly important finding, which is consistent with that observed in other studies reporting an exacerbation of mental disorders in young people, probably related to the wide-ranging effects of the pandemic on this specific age group [18].

Finally, we found that the reduction of ED psychiatric consultations did not homogeneously affect all psychiatric diagnoses. Indeed, the observed reduction affected mainly common mental disorders (such as adjustment disorders, anxiety disorders, depression) and, to less extent, other diagnoses. On the contrary, most severe psychiatric conditions, such as psychoses, had registered an increase in 2021 with respect to 2019 (whereas the number of ED psychiatric consultations for psychosis in 2020 remained substantially stable in comparison to the pre-pandemic year).

The increase of ED psychiatric consultations for psychosis observed in 2021 is an interesting finding. Available literature seems to suggest that there might be an increased occurrence of psychotic episodes in people with established disorders during the COVID-19 pandemic [19–21], probably due to a number of precipitating factors, including prolonged isolation, fear of contagion, presence of pandemic-related conspiracy theories and delusion-like beliefs [19].

It is also interesting that ED consultations for anxiety and mood disorders were consistently reduced over the two years of pandemic, without returning to levels of pre-pandemic year. This finding may be counterintuitive, especially in the light of increased incidence of stress, anxiety and depressive disorders due to the psychological impact of the COVID-19 pandemic on the general population [22–24]. We hypothesize, however, that the increased incidence of common mental disorders in the general population may related to mild or very mild conditions, most of which can be self-managed without necessarily the need for a specialist intervention. Moreover, the fear of contagion might have acted as a deterrent for many people with common mental disorders who, even in highly distressing situations, would have preferred to self-manage their distress rather than take the risk of being infected within crowded emergency departments.

Due to these avoidant and safety behaviours, the lockdown might have paradoxically improved the appropriateness of access to emergency departments by people with anxiety and stress disorders, thus leading to only those patients with severe urgent conditions seeking emergency care. Indeed, the inappropriate use of emergency departments by people with anxiety disorders has been found in some studies, reporting that only half of the anxiety-related ED psychiatric consultations are classified as “urgent” [25, 26]. On the other hand, the fear of contagion may have deterred even those patients with real urgent care needs resulting in worsening of the disorder. Finally, we cannot exclude that the effect on the reduction of ED psychiatric consultations for common mental disorders might be due to a series of initiatives, delivered face-to-face or through an e-health approach (online counselling services, using social media platforms, e-mails or telephone), from public and/or private organizations in Italy aiming to provide psychological support to the general population or within workplaces [27].

This study has several strengths. First, this study considers the whole years 2020 and 2021, rather than limited periods. Second, in addition to annual variations, this study also considered different phases corresponding to different pandemic scenarios. Third, findings were stratified by diagnosis, sex and age groups. Fourth, this study employed an approach to data analysis (i.e. the ITSAs) that made it possible to analyse changes in ED psychiatric consultations during the different phases of the pandemic in statistical terms and not only in terms of graphical displays.

This study also has several limitations. First, ED psychiatric consultations were drawn from one single hospital trust located in northern Italy and thus cannot be considered representative of the national territory. Second, even though Verona Academic Hospital Trust is one of the largest in Italy, the overall number of ED psychiatric consultations is relatively small and thus little variations might have had a larger impact on findings. Third, our study only focused on the adult population and did not consider ED consultations for children and adolescents. Fourth, psychiatric diagnoses were made on clinical basis; this limitation, however, is inherent to the methodology used in this study, as it is well known that routinely collected healthcare data may potentially suffer from low diagnostic accuracy [28]. Finally, the database extracted did not allow us to establish whether people asking for ED psychiatric consultations had a recent onset psychiatric disorder or were patients already in contact with mental health services.

## Conclusions

This observational study found a marked decline of ED psychiatric consultations during the lockdown, the post-lockdown phases and the phases corresponding to the second and third pandemic waves in 2021. However, this reduction impacted exclusively on milder psychiatric conditions (e.g., adjustment, anxiety, stress, and depressive disorders), whereas ED consultations for severe mental disorders (such as psychoses) did not reduce, but rather increased during the second pandemic year. Interestingly, during this period, ED psychiatric consultations also increased for young adults. The increase in emergency consultations for patients with psychosis and for young adults raises concerns on the overall capacity of mental health services in the community to manage and to provide appropriate care to the most vulnerable segments of the population in times of crisis. Mental health services should be prepared to implement alternative outreach strategies, such as telepsychiatry or domiciliary services, to support these populations requiring special attention. Future research should aim to monitor the long-term impact of the pandemic on the mental health system as a whole, by conducting ad hoc longitudinal analyses on ED psychiatric consultations and by correlating the dynamics of emergency psychiatric consultations with the response of mental health services at the community and outpatient level.

## Data Availability

The data used to support the findings of this study are available from the corresponding author upon reasonable request.
